# Highly Efficient Retrograde Gene Transfer into Motor Neurons by a Lentiviral Vector Pseudotyped with Fusion Glycoprotein

**DOI:** 10.1371/journal.pone.0075896

**Published:** 2013-09-24

**Authors:** Miyabi Hirano, Shigeki Kato, Kenta Kobayashi, Tomoaki Okada, Hiroyuki Yaginuma, Kazuto Kobayashi

**Affiliations:** 1 Department of Molecular Genetics, Institute of Biomedical Sciences, Fukushima Medical University School of Medicine, Fukushima, Japan; 2 Section of Viral Vector Development, National Institute of Physiological Sciences, Okazaki, Japan; 3 Department of Neuroanatomy & Embryology, Fukushima Medical University School of Medicine, Fukushima, Japan; 4 Core Research for Evolutional Science and Technology, Japan Science and Technology Agency, Kawaguchi, Japan; University of Pittsburgh School of Medicine, United States of America

## Abstract

The development of gene therapy techniques to introduce transgenes that promote neuronal survival and protection provides effective therapeutic approaches for neurological and neurodegenerative diseases. Intramuscular injection of adenoviral and adeno-associated viral vectors, as well as lentiviral vectors pseudotyped with rabies virus glycoprotein (RV-G), permits gene delivery into motor neurons in animal models for motor neuron diseases. Recently, we developed a vector with highly efficient retrograde gene transfer (HiRet) by pseudotyping a human immunodeficiency virus type 1 (HIV-1)-based vector with fusion glycoprotein B type (FuG-B) or a variant of FuG-B (FuG-B2), in which the cytoplasmic domain of RV-G was replaced by the corresponding part of vesicular stomatitis virus glycoprotein (VSV-G). We have also developed another vector showing neuron-specific retrograde gene transfer (NeuRet) with fusion glycoprotein C type, in which the short C-terminal segment of the extracellular domain and transmembrane/cytoplasmic domains of RV-G was substituted with the corresponding regions of VSV-G. These two vectors afford the high efficiency of retrograde gene transfer into different neuronal populations in the brain. Here we investigated the efficiency of the HiRet (with FuG-B2) and NeuRet vectors for retrograde gene transfer into motor neurons in the spinal cord and hindbrain in mice after intramuscular injection and compared it with the efficiency of the RV-G pseudotype of the HIV-1-based vector. The main highlight of our results is that the HiRet vector shows the most efficient retrograde gene transfer into both spinal cord and hindbrain motor neurons, offering its promising use as a gene therapeutic approach for the treatment of motor neuron diseases.

## Introduction

Motor neuron diseases, including amyotrophic lateral sclerosis and spinal muscular atrophy, are characterized by progressive muscle weakness and paralysis resulting from degeneration of motor neurons in the spinal cord and brain [1-3]. Although the molecular and cellular mechanisms underlying the pathogenesis of motor neuron diseases still remains unknown, mutations in some genes linked to motor neuron death have been found [4-7]. The development of gene therapy technique to introduce transgenes that promote neuronal survival and protection into motor neurons should be an effective approach for the treatment of motor neuron diseases.

Retrograde axonal transport of certain viral vectors confers an advantage for the delivery of genes into neuronal cell bodies situated in regions remote from the injection site. The intramuscular injection of an adenoviral vector enables the delivery of transgenes into motor neurons [8]. This adenovirus-mediated transfer of genes involved in neuronal survival and protection, such as brain-derived neurotrophic factor, ciliary neurotrophic factor, glial cell line-derived neurotrophic factor, and neuronal apoptosis inhibitory protein, prevents motor neuron death in axotomy-induced injury models [9-13]. In addition, the intramuscular injection of adeno-associated virus (AAV) serotype 6 and 9 vectors also deliver desired transgene for gene therapy into motor neurons for gene therapy [14-16].

**Figure 1:**
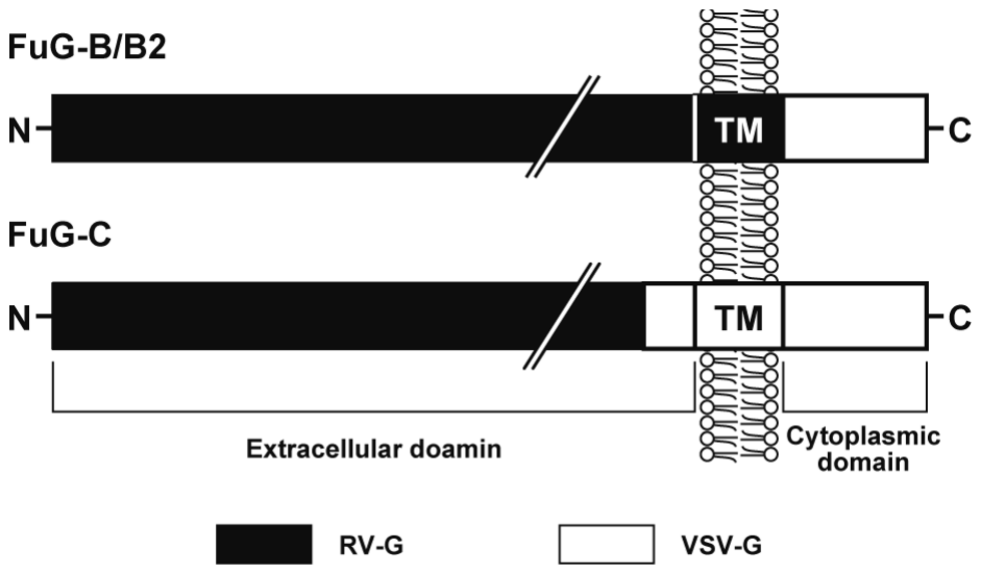
Schematic drawing of viral envelope glycoproteins. FuG-B/B2 consists of the extracellular and transmembrane domains of RV-G fused to the cytoplasmic domain of VSV-G. FuG-C is composed of the N-terminal segment of the extracellular domain of RV-G and the C-terminal segment (16 amino acids) of the extracellular domain and the transmembrane/cytoplasmic domains of VSV-G. TM, transmembrane domain.

The lentiviral vector system permits efficient transfer of genes into neuronal cells and persistent long-term expression of the genes [17-20]. This vector system provides a useful strategy for gene therapy of various neurological and neurodegenerative disorders (for reviews, see 21-23). Pseudotyping of equine infectious anemia virus and human immunodeficiency virus type-1 (HIV-1)-based vectors with selective variants of rabies virus glycoprotein (RV-G) increases the efficiency of retrograde gene transfer into motor neurons after intramuscular injection [24-26]. Lentivirus-mediated gene transfer into motor neuron disease models has the protective effects against neuronal death [27,28].

Recently, we developed a vector with highly efficient retrograde gene transfer (HiRet) in the central nervous system by pseudotyping an HIV-1-based vector with fusion glycoprotein B type (FuG-B), in which the cytoplasmic domain of RV-G was replaced by the corresponding part of VSV-G [29]. The gene transfer efficiency of this HiRet vector was improved by using a variant of FuG-B, termed FuG-B2 [30]. Moreover, we also developed a novel type of vector showing neuron-specific retrograde gene transfer (NeuRet) by pseudotyping the HIV-1 vector with fusion glycoprotein C type (FuG-C), in which the short C-terminal segment of the extracellular domain and transmembrane/cytoplasmic domains of RV-G were substituted with the corresponding regions of VSV-G [31]. (The structure of viral fusion glycoproteins is schematically illustrated in Figure 1.) Both neuronal and glial cells around the injection site are transduced by the HiRet vector, whereas only neuronal cells around this site are transduced by the NeuRet vector [29,31]. Although these two vectors show the high efficiency of retrograde gene transfer into different neuronal populations in the brain, the gene transfer efficiency from the muscles into motor neurons has not been tested.

In the present study, we investigated the efficiency of the HiRet (with FuG-B2) and NeuRet vectors for retrograde gene transfer into motor neurons in the spinal cord and hindbrain in mice following the intramuscular injection and compared it with the efficiency of the RV-G pseudotype of the HIV-1-based vector. Our results demonstrated that the HiRet vector has the most efficient retrograde gene transfer into both spinal cord and hindbrain motor neurons, offering a powerful strategy for gene therapy trials for the treatment of motor neuron diseases.

**Figure 2:**
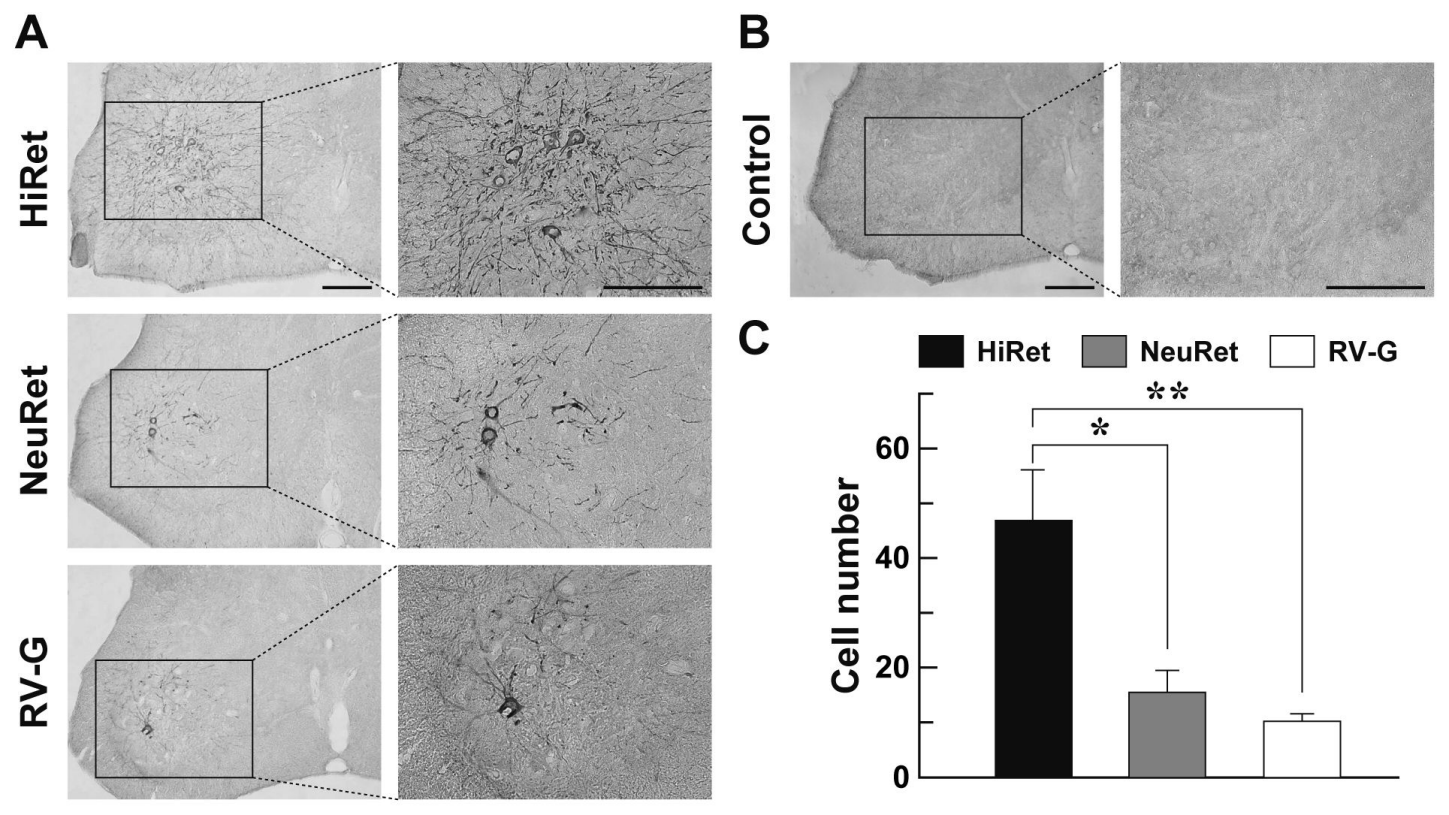
Expression of GFP transgene in the spinal cord through retrograde gene transfer. The HiRet, NeuRet, and RV-G-pseudotyped vectors encoding the GFP transgene with equivalent RNA titers of 5.0 X 10^11^ copies/ml were injected unilaterally into the gastrocnemius muscles of the hindlimb (5.0 μl/site, six sites) of mice. Four weeks later, their spinal cords were processed and a series of sections through the lumbar level were used for immunohistochemistry with anti-GFP antibody. (**A**) Representative images of GFP expression in the lumbar spinal cord. Right images are magnified views of the rectangle areas in the left images. (**B**) Control experiment of GFP immunostaining. Sections through the lumbar spinal cord of the non-injected mice were stained by GFP immunohistochemistry. (**C**) Efficiency of retrograde gene transfer into the lumbar spinal cord motor neurons. The number of GFP-positive cells in the spinal cord was counted. n = 4 for each group. **p < 0.01, *p < 0.05, significant differences from RV-G-pseudotyped and NeuRet vectors, respectively (ANOVA/Bonferroni’s test). Scale bar: 200 µm (**A**,**B**).

## Materials and Methods

### Ethics Statement

All the experiments were conducted in accordance with the guideline of the National Institutes of Health, and the Ministry of Education, Culture, Sports, Science and Technology of Japan, and were approved by the Animal Research Committee of Fukushima Medical University, Fukushima, Japan. We made all efforts to minimize the number of animals used and their suffering.

### Cell Culture

HEK293T cells were obtained from the American Type Culture Collection (ATCC No. CRL-11268, Manassas, VA). The cells were cultured in Dulbecco’s modified Eagle’s medium (Sigma-Aldrich, St. Louis, MO) containing 10% fetal bovine serum (Invitrogen, Tokyo, Japan), 2 mM glutaMAX supplement (Gibco, Tokyo, Japan), and penicillin-streptomycin of 100 units/ml (Gibco) at 37 °C with 5% CO_2_.

### Viral Vector Production

DNA transfection and viral vector preparation were performed as described previously [32,33] with some modifications. The transfer plasmid (pCL20c-MSCV-GFP) contained the cDNA encoding enhanced green fluorescent protein (GFP) downstream of the murine stem-cell virus promoter. The envelope plasmid contained the cDNA encoding FuG-B2 [30], FuG-C [31] or RV-G [34] under the control of the cytomegalovirus enhancer/chicken β-actin promoter [35]. HEK293T cells were transfected with transfer, envelope, and packaging plasmids by the calcium-phosphate precipitation method. Eighteen hr after transfection, the medium was replaced with fresh medium and the cells were incubated for 24 hr. The medium was then harvested and filtered through a 0.45-µm Millex-HV filter unit (Millipore, Billerica, MA). Viral vector particles were pelleted by centrifugation at 6,000 x g for 16-18 hr and resuspended in phosphate-buffered saline (PBS).

**Figure 3:**
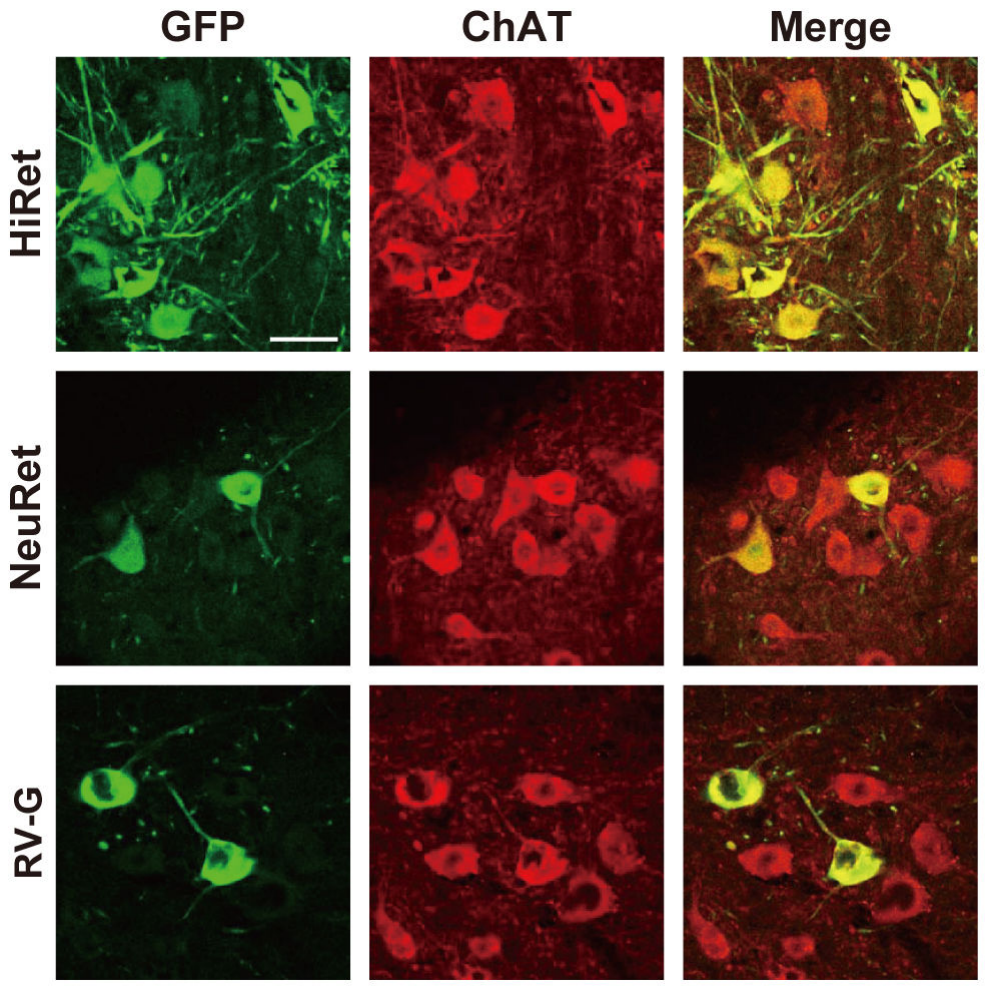
Localization of GFP signals in spinal motor neurons. The HiRet, NeuRet, and RV-G-pseudotyped vectors encoding GFP transgene (5.0 X 10^11^ copies/ml) were injected into the hindlimb muscles (5.0 μl/site, six sites) of mice. Four weeks later, their spinal cords were processed and sections through the lumbar level were then stained by double immunofluorescence histochemistry for GFP and ChAT. GFP-positive, ChAT-positive, and doubly positive cells are shown as green, red, and yellow, respectively. Scale bar: 50 µm.

### Vector Titration

Viral RNA in the vector stock solution was isolated with a NucleoSpin RNA virus kit (Clontech, Mountain View, CA), and the copy number of the RNA genome was determined by using a Lenti-X qRT-PCR titration kit (Clontech) according to the manufacture’s instruction. Viral RNA was reverse-transcribed at 42°C for 20 min, and quantitative PCR was carried out on duplicate samples by using a StepOne real-time PCR system (Applied Biosystems, Tokyo, Japan) under the following conditions: 1 cycle of 95°C for 3 min followed by 40 cycles of 95°C for 15 sec and 60°C for 30 sec. The standard curve was prepared on the basis of serial dilutions of viral RNA control template ranging from 10^5^ to 10^8^ copies.

### Intramuscular Injection

Twenty-eight C57BL/6J mice (3 weeks old) were used for the present study. For injection of viral vectors, mice were anesthetized with sodium pentobarbital (50 mg/kg i.p.) and the vectors were introduced into the gastrocnemius muscles of the right hindlimb (5.0 μl/site, six sites) or into both sides of the tongue lateral and lingual muscles (2.0 μl/site, four sites) through a glass microinjection capillary connected to a Hamilton syringe. There were no cases of early mortality and all animals survived until the time of evaluation (4 weeks postinjection).

#### Histological Analysis

Animals were anesthetized with sodium pentobarbital (50 mg/kg i.p.) and perfused transcardially with 4% paraformaldehyde in 0.1 M phosphate buffer (pH 7.4). Perfusion was carried out 4 weeks after the injection of lentiviral vectors. For immunostaining by the avidin-biotin-peroxidase complex method, transverse sections (30-µm thickness) were incubated with rabbit polyclonal antibody for GFP (Molecular Probes, Eugene, OR) at a 1:2,000 dilution, and then with biotinylated donkey anti-rabbit IgG antibody (Vector Laboratories, Burlingame, CA) at a 1:500 dilution. The immunoreactive signals were visualized with a Vectastain Elite ABC kit (Vector Laboratories). For double immunofluorescence histochemistry, sections were incubated with rabbit polyclonal anti-GFP antibody (1:2,000 dilution) and mouse monoclonal antibody for choline acetyltransferase (ChAT) (1:1000 dilution, Millipore). Sections were then incubated with fluorescein isothiocyanate-conjugated goat anti-rabbit IgG and Cy3-conjugated goat anti-mouse IgG (1:500 dilution, Jackson ImmunoResearch Laboratories, West Grove, PA). Fluorescent images were taken with a confocal laser-scanning microscope (LSM510, Zeiss, Thornwood, NY) equipped with proper filter cube specifications for fluorescein isothiocyanate and Cy3 fluorescence channels.

### Cell Counts

A series of sections through the lumbar segments L3/L4 of the spinal cord and the medulla oblongata were used for immunostaining by the avidin-biotin-peroxidase complex method. The number of immunostained cells in the lumbar spinal cord and the medulla oblongata was counted in every five and two sections, respectively; and the total number of stained cells in each region was calculated.

### Statistical Analysis

For statistical comparisons, the analysis of variance (ANOVA), the *post-hoc* Bonferroni’s test, and Student’s *t* test were used with significant set at *p* < 0.05. All values were expressed as the mean ± SEM of the data.

**Figure 4:**
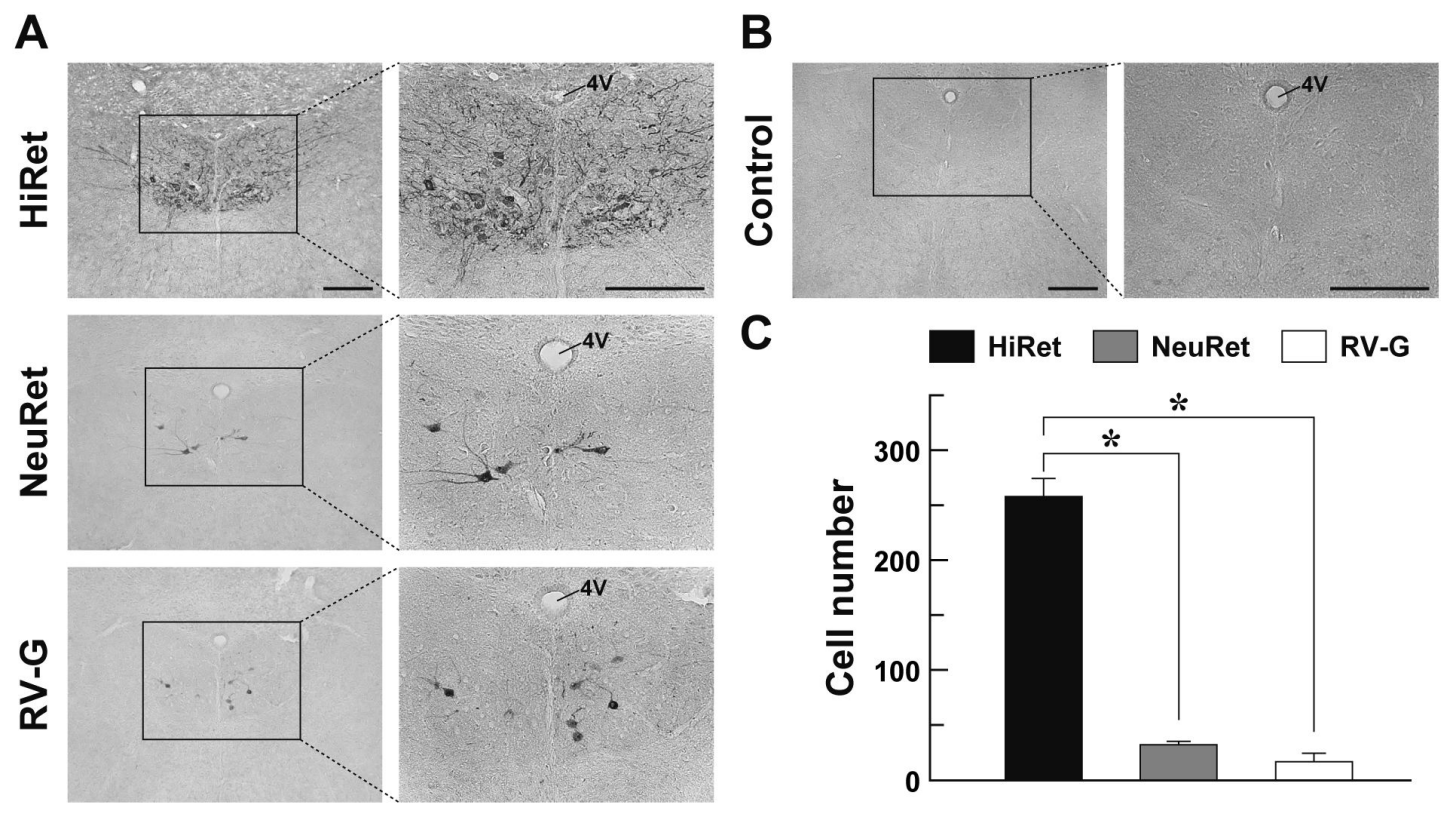
GFP transgene expression in the hindbrain via retrograde gene delivery. The HiRet, NeuRet, and RV-G-pseudotyped vectors encoding the GFP transgene with equivalent RNA titers of 5.0 X 10^11^ copies/ml were injected into the tongue muscles (2.0 μl/site, four sites) of mice. Four weeks later, their brains were processed and a series of sections through the hindbrain were then used for immunostaining with anti-GFP antibody. (**A**) Representative images of GFP expression in the hypoglossal nucleus. Right images are magnified views of the rectangles areas in the left images. (**B**) Control experiment of GFP immunostaining. Sections through the hindbrain of the non-injected mice were stained by GFP immunohistochemistry. (**C**) Efficiency of retrograde gene transfer into the hypoglossal neurons. The number of GFP-positive cells in the hypoglossal nucleus was counted. n = 4 for each group. *p < 0.001, significant difference from the RV-G-pseudotyped or NeuRet vector (ANOVA/Bonferroni’s test). 4V, fourth ventricle. Scale bar: 200 µm (**A**,**B**).

## Results

### Retrograde Gene Transfer into Spinal cord Motor Neurons

To compare the efficiency of retrograde gene transfer into motor neurons after intramuscular injection, we prepared the HiRet vector with FuG-B2 and NeuRet vector with FuG-C, together with the RV-G pseudotype of the HIV-1-based vector. We first injected these vectors encoding the GFP transgene with equivalent RNA titers of 5.0 X 10^11^ copies/ml (5.0 µl X six sites) unilaterally into the hindlimb muscles in mice at the age of 3 weeks. Four weeks later, the spinal cord was processed and sections through its lumbar level were stained by immunohistochemistry with anti-GFP antibody. Many GFP-positive cells with dense nerve fibers were visualized in the ventral horn of the spinal cord in the HiRet vector-injected mice, whereas a small number of immunopositive cells were seen in the corresponding region in the NeuRet or RV-G vector-injected animals (Figure 2A). GFP staining with sections prepared from the non-injected control mice did not show any immunopositive signals in their spinal cord (Figure 2B). To confirm the retrograde gene transfer into spinal cord motor neurons, we performed double immunofluorescence histochemistry for GFP and the motor neuronal marker choline acetyltransferase (ChAT). GFP expression was observed in ChAT-positive neurons in the spinal cord in the mice that had received the intramuscular injection (Figure 3), indicating the retrograde delivery of the GFP transgene into these motor neurons.

To quantitatively evaluate the extent of retrograde gene transfer, we counted the number of GFP-positive cells in the spinal cord and compared them among the HiRet, NeuRet, and RV-G vectors (Figure 2C). The number of cells in the HiRet vector-injected mice (47.0 ± 9.1) was significantly greater as compared with the number in the RV-G vector-injected controls (10.3 ± 1.3) (one way ANOVA, *F*_(2, 9)_ = 11.85, *p* < 0.01; Bonferroni’s test, *p* < 0.01), showing the increases of 4.6-fold. The cell number in the HiRet vector-injected mice was also larger than the value in the NeuRet vector-injected animals (15.5 ± 4.0) (Bonferroni’s test, *p* < 0.05). However, the cell number was not significantly different between the NeuRet and RV-G vector-injected animals (Bonferroni’s test, *p* = 1.00). These data indicate that the HiRet vector gave the highest efficiency of retrograde gene transfer into the spinal cord motor neurons after intramuscular injection.

### Retrograde Gene Delivery into Hindbrain Motor Neurons

**Figure 5:**
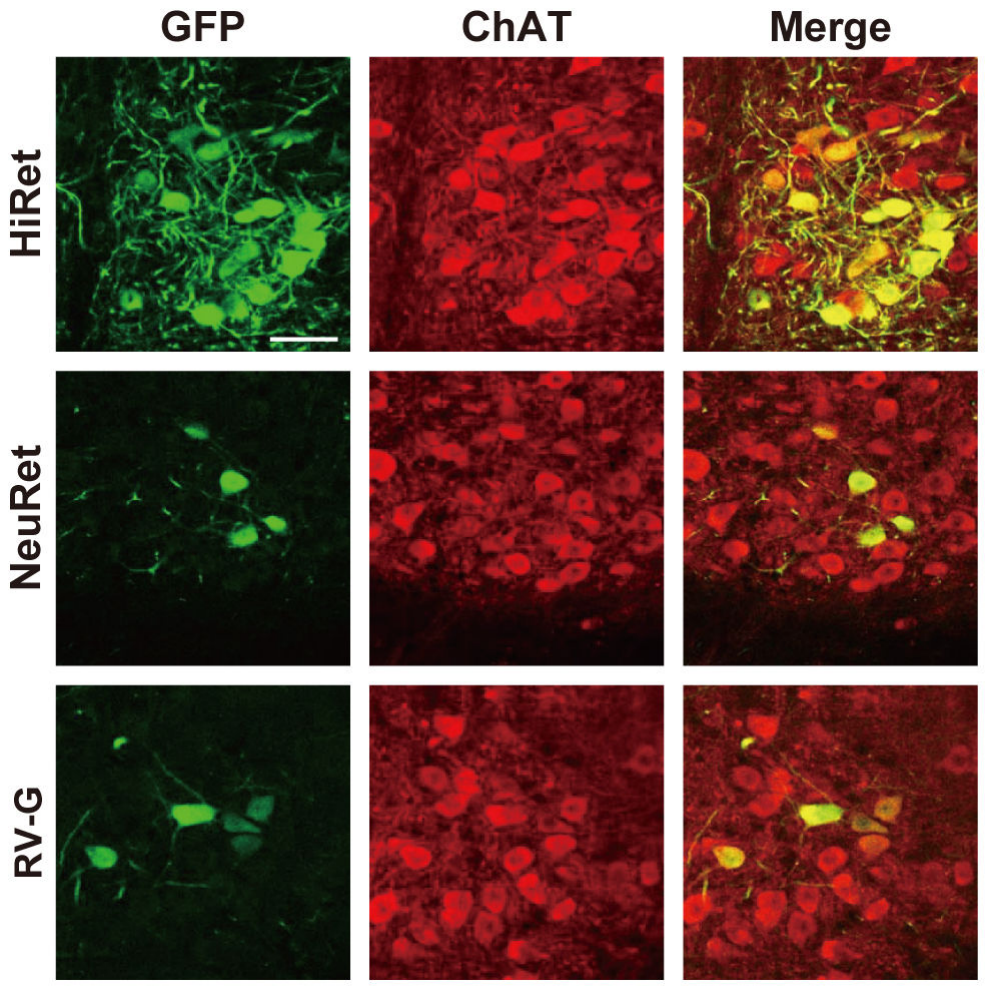
Localization of GFP signals in hindbrain motor neurons. The HiRet, NeuRet, and RV-G-pseudotyped vectors encoding the GFP transgene (5.0 X 10^11^ copies/ml) were injected into the tongue muscles (2.0 μl/site, four sites) of mice. Four weeks later, their brains were processed and sections through the hindbrain were then stained by double immunofluorescence histochemistry for GFP and ChAT. GFP-positive, ChAT-positive, and doubly positive cells are shown as green, red, and yellow, respectively. Scale bar: 50 µm.

To further validate retrograde gene transfer into motor neurons after intramuscular injection, we injected the HiRet, NeuRet, and RV-G vectors encoding the GFP transgene with the same vector concentration as indicated above (2.0 µl X four sites) into the tongue muscles in 3-week-old mice. Four weeks later, their brains were processed and sections through the hindbrain were stained by GFP immunohistochemistry. A number of GFP-positive cells were detected in the hypoglossal nucleus of the posterior hindbrain in the HiRet vector-injected mice, whereas the number of immunopositive cells was smaller in the corresponding nucleus in the NeuRet or RV-G vector-injected animals (Figure 4A). GFP staining with sections prepared from the non-injected control mice showed no immunopositive signals in their brain regions (Figure 4B). Double immunofluorescence histochemistry for GFP and ChAT confirmed the GFP expression in hypoglossal motor neurons in the hindbrain of the mice that had been given the intramuscular injection (Figure 5).

The number of GFP-positive cells in the hindbrain was counted and compared among the three kinds of vectors (Figure 4C). The cell number in the HiRet vector-injected mice (258.5 ± 16.0) was remarkably increased to 14.8-fold of the value in the RV-G vector-injected control mice (17.5 ± 6.6) (one way ANOVA, *F*_(2, 9)_ = 178.87, *p* < 0.001; Bonferroni’s test, *p* < 0.001). The cell number in the HiRet vector-injected mice was also increased significantly as compared with that in the NeuRet-injected animals (32.3 ± 2.8) (Bonferroni’s test, *p* < 0.001), although the number was not indistinguishable between the NeuRet and RV-G vector-injected animals (Bonferroni’s test, *p* = 0.986). Therefore, the HiRet vector displayed the highest efficiency of retrograde gene delivery not only into the spinal cord motor neurons but also into the hindbrain motor neurons following intramuscular injection.

**Figure 6:**
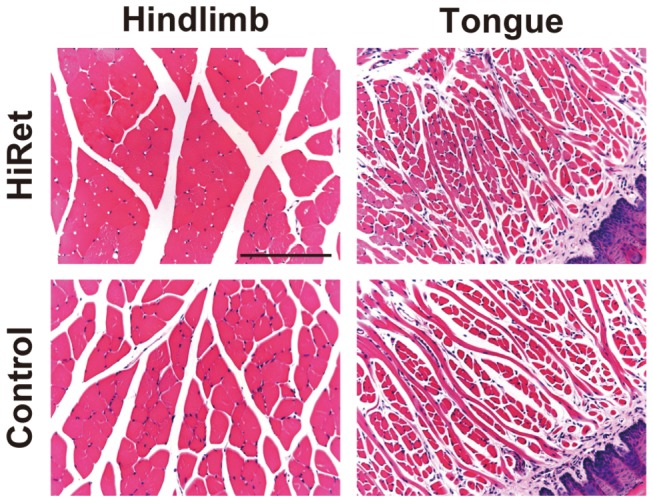
Morphology of muscle tissues after intramuscular injection of the viral vector. The HiRet vector encoding the GFP transgene (5.0 X 10^11^ copies/ml) was injected into the gastrocnemius muscles of the hindlimb (5.0 μl/site, six sites) or the tongue muscles (2.0 μl/site, four sites) of mice. Four weeks later, their hindlimb and tongue were processed and sections were stained with hematoxylin and eosin. Sections through the muscles prepared from the non-injected mice were used for the control experiments. Scale bar: 200 µm.

To test whether there are damages in the muscle tissues after intramuscular injection of the viral vectors, we prepared sections through the hindlimb and tongue muscles from the HiRet-vector injected mice and stained them with hematoxylin and eosin. The staining showed normal muscle fiber structure with no signs of inflammatory responses in these tissues by vector injection (Figure 6).

## Discussion

In the present study, we examined the efficiency of the HiRet and NeuRet vectors for retrograde gene transfer into motor neurons in the spinal cord and hindbrain in mice after intramuscular injection and compared it with the efficiency of the RV-G pseudotyped vector. Our results highlighted that the HiRet vector shows the capability for the most efficacious retrograde transfer of the transgene into both spinal cord and hindbrain motor neurons.

For the HiRet vector preparation, we used FuG-B2, which is a variant of FuG-B and its extracellular domain was derived from the Pasteur virus strain of rabies virus [30]. In contrast, the extracellular domain (N-terminal segment) of FuG-C is derived from the challenge virus strain of rabies virus [31]. The amino acid sequence of the RV-G extracellular domain shows a 94% homology between the Pasteur virus and challenge virus standard strains. There is the possibility that a slight difference in amino acid sequence between these two strains may affect the efficiency for retrograde gene transfer into motor neurons. However, we preliminarily tested the gene transduction of the NeuRet vector having a variant of FuG-C, in which the RV-G segment was replaced by the corresponding part derived from the Pasteur virus strain, and found no significant difference from the original FuG-C vector in terms of the efficacy of retrograde gene transduction into the hypoglossal motor neurons after intramuscular injection (see Figure S1). Thus, the enhanced retrograde gene transfer efficiency into motor neurons by the HiRet vector may be attributable to the difference in the type of fusion envelope glycoproteins, but not to the sequence variation between the rabies virus strains.

Previous reports indicate that rabies virus interacts with certain neuronal receptors, such as the nicotinic acetylcholine receptor, low-affinity nerve growth factor receptor, and neural cell adhesion molecule [36-39]. Especially, *in vitro* experiments show that rabies virus binds to the nicotinic acetylcholine receptor α-subunit at the neuromuscular junctions [40,41], suggesting that this binding may be secondarily involved in the uptake of viral particles into nerve terminals [42]. The structural feature of FuG-B2 may facilitate the interaction with this putative receptor at the neuromuscular junctions, resulting in the enhanced retrograde gene delivery into motor neurons. The difference in fusion glycoprotein types is also known to influence the efficiency of retrograde gene delivery into distinct neuronal pathways in the mouse brain [43].

Various serotypes of AAV vectors have been used for gene delivery into motor neurons of animal models, but AAV serotypes that show efficient retrograde gene transfer are limited. A previous report indicated retrograde gene transfer of AAV serotype 6 into spinal cord motor neurons after injection into the hindlimb gastrocnemius, and the extent of gene transfer was ~50 motor neurons in mice [15]. The transfer efficiency in their vector system attained to the plateau above 2 X 10^7^ transducing units of the vector. In the present study, our HiRet vector also showed similar transduction efficiency into spinal cord motor neurons, but increasing titer of the HiRet vector may improve the efficiency of retrograde gene transfer. AAV serotype 9 showed retrograde gene transfer into hypoglossal neurons after injection into the tongue muscles, showing the extent of gene transfer of ~230 hypoglossal motor neurons [16]. Our HiRet vector appeared to display more efficient retrograde gene transfer into hypoglossal motor neurons (approximately 260 motor neurons). In addition, a lentiviral vector system allows persistent long-term expression of transgene [17-20]. The packaging capacity of AAV vectors is considered to be restricted to approximately 4.7 kilobases [44,45], whereas lentiviral vectors possess greater insertional size of transgene [46]. Therefore, our HiRet vector system shares various merits towards the application of retrograde gene transfer into motor neurons by the intramuscular injection.

Retrograde axonal transport of viral vectors provides great advantages in model experiments for gene therapy of motor neuron diseases. Actually, the intramuscular injection of adenoviral, adeno-associated viral, and RV-G-pseudotyped lentiviral vectors permits gene delivery into target neurons in animal models of motor neuron diseases [9-13,27,28], although some trials suggest the ineffectiveness of gene delivery into simple neuronal populations to generate phenotypic improvement [15]. Here, we demonstrated that the HiRet vector achieved the most prominent retrograde transfer of the gene into both spinal cord and hindbrain motor neurons in rodents. Motor neurons were transduced by the HiRet vector with a considerably greater efficiency than the standard lentiviral vector pseudotyped with RV-G. Our newly-developed gene transfer technique will promote the further development of genetic, therapeutic approaches for the treatment of intractable motor neuron diseases.

## Supporting Information

Figure S1 **Comparison of retrograde gene transfer efficiency between the NeuRet vectors with FuG-C and its variant.** The NeuRet vectors with FuG-C or a variant of FuG-C (FuG-C/PV) encoding the GFP transgene with equivalent copy numbers of viral RNA (5.0 X 10^11^ copies/ml) were injected into the tongue muscles (2.0 μl/site, four sites) of mice. Four weeks later, sections through the hypoglossal nucleus in the hindbrain were used for immunohistochemistry with anti-GFP antibody. (**A**) Representative images of GFP expression pattern in the hindbrain region. (**B**) Number of GFP-positive cells in the hypoglossal nucleus. n = 3 for each group. The cell number did not show any significant difference between FuG-C and FuG-C/PV (Student’s *t* test, *p* = 0.371). 4V, fourth ventricle. Scale bar: 200 µm.
(TIF)
