# Supplementary figures and images for: Highly Efficient Retrograde Gene Transfer into Motor Neurons by a Lentiviral Vector Pseudotyped with Fusion Glycoprotein
Source: PLoS One. 2013 Sep 24;8(9):e75896. doi: 10.1371/journal.pone.0075896 (PMC3782444; doi:10.1371/journal.pone.0075896)

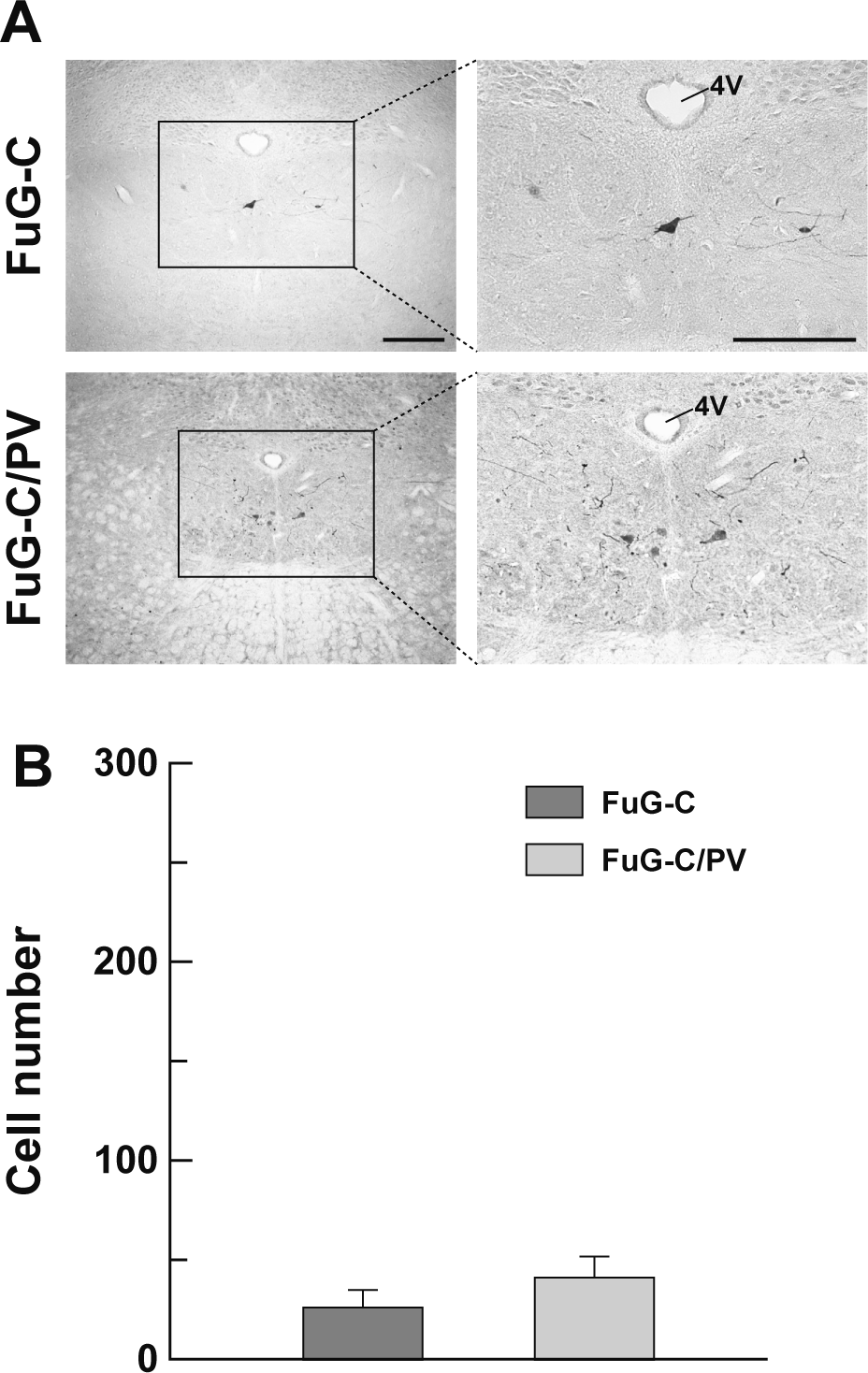

Supplement: Figure S1 — Comparison of retrograde gene transfer efficiency between the NeuRet vectors with FuG-C and its variant. The NeuRet vectors with FuG-C or a variant of FuG-C (FuG-C/PV) encoding the GFP transgene with equivalent copy numbers of viral RNA (5.0 X 1011 copies/ml) were injected into the tongue muscles (2.0 μl/site, four sites) of mice. Four weeks later, sections through the hypoglossal nucleus in the hindbrain were used for immunohistochemistry with anti-GFP antibody. (A) Representative images of GFP expression pattern in the hindbrain region. (B) Number of GFP-positive cells in the hypoglossal nucleus. n = 3 for each group. The cell number did not show any significant difference between FuG-C and FuG-C/PV (Student’s t test, p = 0.371). 4V, fourth ventricle. Scale bar: 200 µm. (TIF) [file pone.0075896.s001.tif]
